# Genetic dissection of blood lipid traits by integrating genome-wide association study and gene expression profiling in a porcine model

**DOI:** 10.1186/1471-2164-14-848

**Published:** 2013-12-03

**Authors:** Congying Chen, Bin Yang, Zhijun Zeng, Hui Yang, Chenlong Liu, Jun Ren, Lusheng Huang

**Affiliations:** Key Laboratory for Animal Biotechnology of Jiangxi Province and the Ministry of Agriculture of China, Jiangxi Agricultural University, 330045 Nanchang, China

**Keywords:** Blood lipids, Genome-wide association study, Gene expression, Candidate gene, Pig

## Abstract

**Background:**

Serum concentrations of total cholesterol (TC), low-density lipoprotein cholesterol (LDL-C), high-density lipoprotein cholesterol (HDL-C) and triglycerides (TG) are highly heritable traits that are used clinically to evaluate risk for cardiovascular disease in humans. In this study, we applied a genome-wide association study (GWAS) in 1,075 pigs from two populations and gene expression studies on 497 liver samples to dissect the genetic basis of serum lipids in a pig model.

**Results:**

We totally identified 8, 5, 2 and 3 genomic loci harboring 109 SNPs that were significantly associated with LDL-C, TC, TG and the ratio of HDL-C/LDL-C in two experimental populations, respectively. In the F_2_ population, the most prominent SNP was identified at the SSC3: 124,769,847 bp where *APOB* is the well-known candidate gene. However, in the Sutai population, the most number of significant SNPs was identified at SSC2: 64.97-82.22 Mb where *LDLR* was identified as the candidate gene. Furthermore, we firstly reported 4 novel genomic loci in pigs harboring the LDL-C-associated SNPs. We also observed obvious population heterogeneity in the two tested populations. Through whole-genome gene expression analysis, we detected 718 trait-correlated expressions. Many of these transcripts correspond to candidate genes for blood lipids in humans. The GWAS mapped 120 *cis-*eQTLs and 523 *trans-*eQTLs for these transcripts. One gene encoding the transcript gnl|UG|Ssc#S35330332 stands out to be an important candidate gene for LDL-C by an integrative analysis of GWAS, eQTL and trait-associated expression.

**Conclusions:**

We identified the genomic regions or candidate genes associated with blood lipids by an integrative analysis of GWAS, QTT and eQTL mapping in pigs. The findings would benefit the further identification of the causative genes for blood lipid traits in both pigs and humans.

**Electronic supplementary material:**

The online version of this article (doi:10.1186/1471-2164-14-848) contains supplementary material, which is available to authorized users.

## Background

Blood lipids are transported in the bloodstream of human and animal. They are important indicators for whole body lipid metabolism. The screening tests of blood lipid concentrations of total cholesterol (TC), low-density lipoprotein cholesterol (LDL-C), high-density lipoprotein cholesterol (HDL-C) and triglycerides (TG) are used clinically to evaluate the risk for cardiovascular diseases and to give the guidance for prescription of medicine. Serum concentrations of blood lipids are highly heritable phenotypes [1]. To date, a number of genome-wide association studies (GWAS) have successfully identified over 100 loci associated with one or more blood lipid traits in humans [2]. For instance, a GWAS in > 100,000 individuals of European ancestry revealed 95 significant loci associated with plasma lipids [3]; a GWAS for 22 plasma lipoprotein traits identified 43 loci associated with plasma lipoprotein size, concentration and cholesterol content in 17,296 women from the Women’s Genome Health Study [4]. Further investigations of three loci have identified *GALNT2* [5], *TRIB1* [6], and *SORT1* [7] as causative genes for blood lipid traits. However, these loci explain only a small proportion of trait variability, suggesting that many determinants remain unexplored.

The pig is an important biomedical model [8]. Compared to humans, pigs not only have similar lipoproteins but also show similar morphology and biochemical composition in atherosclerosis plaque [9]. Moreover, the advantages of pig as a biomedical model for blood lipids also include: I. pigs can be raised in a unified and standard condition; II. Large-scale RNA samples of liver are easily available for gene expression analysis. Recently, a number of quantitative trait loci (QTL) have been mapped for porcine blood lipids using the whole genome-linkage analysis. To date, 18 QTL for TC, 19 for HDL-C, 11 for HDL-C/LDL-C, 12 for LDL-C, and 21 for TG have been reported in the pig QTL database [10]. However, no causative gene has been identified for these QTL. The rare *LDL receptor* (*LDLR*) mutations contribute to an autosomal recessive hypercholesterolemia in the specific pig strains [11, 12], and *apolipoprotein B* (*APOB*) mutations are associated with elevated plasma cholesterol and atherosclerosis in pigs in relation to atherosclerosis [13, 14]. But none of them seems to be the causal mutation but probably represent closely linked polymorphisms to the QTL of serum cholesterol and triglycerides concentrations in a half-sib Duroc pig population [15].

The GWAS is a powerful tool to identify genomic regions affecting phenotypic traits but not efficient for identifying causative mutations [16]. The application of global gene expression analysis has provided a wealth of data relevant to complex traits. For example, Ponsuksili et al. identified 663 genes with fatness-associated expression in porcine liver and mapped their expression quantitative trait loci (eQTL) [17]. More recently, a number of studies have shown that an integrative analysis of GWAS, eQTL and bionetwork can facilitate the identification of causative mutations leading to changes of phenotypes [18, 19], For instance, Schadt et al. identified *SORT1* and *CELSR2* as candidate susceptibility genes for LDL-C using the integrative approach [19]. Wimmer et al. characterized *AHNAK*, *SLC3A2* and *MAP4K4* as candidate genes for meat drop loss by integrating data of gene expression, eQTL and phenotypic QTL [20].

In this study, a GWAS for porcine blood lipid traits was conducted in two populations including a White Duroc × Erhualian F_2_ intercross and a Chinese synthesized line (Sutai pigs). Genome-wide gene expression and quantitative trait transcript (QTT) analyses as well as eQTL mapping were also performed to facilitate the identification of candidate genes for these traits. This study provides useful information for the genetic architecture of blood lipids and for human cardiovascular diseases.

## Methods

### Experimental populations

All samples in this study were from the White Duroc × Erhualian F_2_ resource population and Sutai pigs. The White Duroc × Erhualian F_2_ resource population was constructed as described previously [21]. In brief, 2 White Duroc boars and 17 Erhualian sows were mated to produce F_1_ animals, and then 9 F_1_ sires and 59 F_1_ dams were randomly intercrossed, avoiding full-sib mating, to generate 1,912 F_2_ individuals. Sutai pigs were synthetized from Duroc × Erhualian crossing through selection of 18 generations. A total of 435 Sutai pigs from 5 boar families were used in this study. The animals were raised in a standard indoor condition with natural lighting and were fed three times a day using the feed containing 16% of crude protein, 3100 kJ of digestible energy, and 0.78% of lysine. Water was available *ad libitum* from nipple drinkers. These animals were slaughtered at 240 ± 3 days after fasting but water-free overnight (about 12 hours). All samples were collected according to the guidelines for the care and use of experimental animals established by the Ministry of Agriculture of China. Animal Care and Use Committee (IACUC) in Jiangxi Agricultural University specifically approved this study.

### Phenotype recording

Blood samples were collected from the major artery serum vessels near the heart when the animals were exsanguinated. After coagulation at room temperature, the clots were centrifuged at 3000 rpm at 4°C for 20 min to separate serums. All serum samples were then stored at -80°C until utilized. LDL-C, HDL-C, TG and TC levels for 760 F_2_ animals (411 males and 349 females) and 435 Sutai pigs (228 males and 207 females) were measured by direct assay with diagnostic kits of Determiner-L LDL-C, Determiner-L HDL-C, Determiner-L TCII and Determiner-C TG (Kyowa Medex, Japan), respectively, following the manufacturer’s instructions. All measurements were performed in an AU5421 Automatic Biochemistry Analyzer (Backman-Kelt, USA) at the First Affiliated Hospital of Nanchang University.

### RNA extraction

Liver samples were harvested from 497 F_2_ animals for RNA isolation within 30 min after slaughter. The tissues were put into the sterile and frozen cryopreservation tubes and dipped into liquid nitrogen, and then conserved in -80°C ultra freezer until RNA extraction. Total RNA was isolated with TRIzol (Invitrogen, USA) following the manufacture’s instruction. The residual DNA was cleared away from total RNA with RNase-free DNase I (New England Biolabs, UK) for 30 min at 37°C. The quality of total RNA was assessed by a 2100 Bioanalyzer (Agilent, UK) and agarose gel electrophoresis.

### SNP genotyping and GWAS analysis

All animals were genotyped using Porcine SNP60 BeadChips according to the *Infinium HD Assay Ultra* protocol (Illumina Inc., USA). The positions of all 62,163 SNPs from the Porcine SNP60 BeadChip on the current pig genome assembly (Sscrofa 10.2) were retrieved from the NRSP-8 Community Data Repository [22]. The quality control (QC) of genotypes was performed with GenABEL procedure in R. The SNPs with call rate < 95%, or minor allele frequency < 5%, or Hardy Weinberg equilibrium (HWE) *P*-value < 5 × 10^-6^, or the X-linked SNPs that were likely to be autosomal (odds > 1000) were excluded from further analysis. Finally, total 39,454 and 45,322 SNPs passed the quality control in the White Duroc × Erhualian F_2_ resource population and the Sutai pigs, respectively.

The associations of the filtered SNPs with serum lipid levels were evaluated using a mixed model based on the score test approach [23]. The model included a random polygenic effect for which the variance-covariance matrix was proportional to genome-wide identity-by-state. The model equation is: *Y = Xb + Sα + Zu + e*, where *Y* is the vector of phenotypes, *b* is the vector of fixed effects including sex and batch, *α* is the vector of the SNP substitution effect and *u* is the random additive genetic effects with *u* ~ N (0, **G**σ_α_^2^), where G is the genomic relationship matrix that was constructed based on SNP markers, and σ_α_^2^ is the polygenetic additive variance. *e* is a vector of residual errors with a distribution of N (0, *I*σ_e_^2^), where *I* is the identity matrix and σ_e_^2^ is the residual variance. X, S and Z are the incidence matrices for *b*, *α* and *u*. The analysis was conducted by mmscore function with GenABEL in R package. Bonferroni correction was used to adjust the multiple tests. A conservative threshold of *P* < 0.05/SNP number was applied for genome-wide significance, and 1/SNP number was set as suggestive significance. Quantile-quantile (Q-Q) plots and Manhattan plots were drawn using the corresponding *P* values by R package. We calculated the phenotypic variations explained by the top significant SNPs using (V _reduce_ – V _full_)/V _reduce_, where V _full_ and V _reduce_ are residual variances of the models for association analysis with and without SNP term, respectively. We also searched candidate genes with functional relevance to serum lipids or lipid metabolism in an interval of 5.0 Mb centered at the top SNP at each significant locus.

### QTT analysis and eQTL mapping

Digital gene expression (DGE) analyses of genome-wide transcripts were performed on 497 F_2_ liver samples as described previously [24]. In brief, mRNA was isolated from total RNA with the magnetic oligo (dT) beads (invitrogen, USA). Using the mRNA attached to the bead as a template, double-stranded cDNA was synthesized with oligo-d (T) primers, and then digested with restriction enzymes *Nla* III and *Mme* I (New England Biolabs, UK). The digested-cDNA was ligated to Illumina specific adapters 1 and 2. Polymerase chain reaction (PCR) was performed to enrich the cDNA library with two primers that annealed to the ends of the adapters. After purification and denaturation, the single chain molecules of each cDNA library were loaded onto the flowcell and sequenced on a GA II sequencer (Illumina, USA).

Tag data sets were analyzed according to the BGI bioinformatics protocols for digital gene expression. Briefly, the raw tags were firstly filtered to produce the clean tag data. To map the clean tags to reference transcript sets or to the pig reference genome, the reference transcript sets were downloaded from the database of PEDE [25] and pig unigene in NCBI [26]. The redundant transcripts which overlapped between the two databases were discarded. The virtual libraries containing all the possible 17 base-length sequences of these reference transcripts next to an *Nla* III restriction site were created using in-house Perl scripts. The clean tag sequences were then mapped using SOAP2 that allowed up to one mismatches in 21-bp tag sequences [27]. The expression profile of each transcript was defined as the number of clean tags that could be uniquely mapped to the reference transcript sequence. Then, the expression levels were normalized to TPM (number of tags mapped to each gene per million clean tags).

The gene expression profiles and phenotypic data were further adjusted for gender, batch and kinship using a robust linear regression model. Associations of gene expression level with serum blood lipid concentrations were evaluated with spearman coefficient by R software. To adjust the multiple tests, a conservative *P* < 0.0005 was set as threshold. eQTL mapping was performed for those trait-correlated transcripts using mixed linear model implemented by *mmscore* function of GenABEL in R package. Sex and batch were considered as fixed effects, the genetic co-variances among samples were also taken into account by fitting kinship matrix derived from genotypes of whole-genome SNP markers. A bonferroni correction was applied to adjust the multiple tests. All the above mentioned analyses were carried out with R package.

## Results

The phenotypic characteristics of qualified samples in the two experimental populations are summarized in Table 1. Both populations had comparably average values of serum lipid contents. We estimated the narrow-sense heritability (*h*^*2*^) for each of the 5 serum lipid traits in the two pig populations. A medium to high *h*^*2*^ (0.31-0.71) was observed for these traits, suggesting considerable genetic contribution to serum lipid contents in pigs (Table 1).

**Table 1:** **Summary statistics for serum lipid traits in the tested samples**

Trait	White Duroc × Erhualian F_2_intercross			Sutai		
	N	mean ± SD	*h* ^2*^	N	mean ± SD	*h* ^*2*^
LDL-C	682	1.31 ± 0.27	0.48	393	1.27 ± 0.27	0.71
HDL-C	681	1.13 ± 0.23	0.37	393	1.37 ± 0.29	0.63
HDL-C/LDL-C	681	0.90 ± 0.21	0.44	393	1.11 ± 0.26	0.68
TG	682	0.28 ± 0.15	0.43	393	0.27 ± 0.12	0.31
TC	654	2.67 ± 0.43	0.47	393	2.34 ± 0.36	0.70

*h ^*2*^, heritability estimates

### GWAS for blood lipids in the White Duroc × Erhualian F_2_ resource population

We performed GWAS for 5 serum lipid traits in the White Duroc × Erhualian F_2_ resource population using an additive model. The final numbers of animals passed the QC are listed in Table 1. Quantile-quantile (Q-Q) plots of observed *P*-values for single SNP association tests are shown in Additional file 1: Figure S1. Except for the tail likely indicating true associations, the distributions of observed *P*-values did not deviate from null distribution, which rules out systematic bias due to bad genotyping or population substructure. At a suggestive significance threshold of *P* < 2.53 × 10^-5^ (1/39,454), 22 SNPs corresponding to 5 chromosomal regions were significantly associated with one or more phenotypes (Additional file 2: Figure S2 and Additional file 3: Table S1A). Of them, only 6 associations on SSC1 and SSC3 achieved the genome-wide significance level (*P* < 1.27 × 10^-6^) (Table 2). The most prominent association was identified at SSC3: 124,679,847 bp.

**Table 2:** **Summary of the chromosomal regions associated with blood lipids at the genome-wide significance level**

Trait	population	Top SNPs	Num. of SNP^***a***^	Position (bp)^***b***^	Phenotypic variance (%)^***c***^	P-value	candidate gene^***d***^
TC	F_2_	ASGA0016328	2	SSC3: 124,769,847	7.60	2.03 × 10^-7^	*APOB*
	Sutai	MARC0101455	3	SSC2: 56,469,735	9.75	4.40 × 10^-8^	*CRTC1*, *CERS1*
	Sutai	DIAS0002715	4	SSC2: 70,246,409	8.83	5.76 × 10^-8^	*SMARCA4*, *LDLR*
	Sutai	ALGA0109254	1	SSC5: 3,550,340	13.34	2.05 × 10^-7^	Undetected
LDL-C	F_2_	ASGA0090960	1	SSC1: 63,541,683	11.66	2.69 × 10^-8^	Undetected
	F_2_	ASGA0016328	3	SSC3: 124,769,847	9.80	1.90 × 10^-10^	*APOB*
	Sutai	M1GA0002939	15	SSC2: 60,318,677	16.27	8.27 × 10^-11^	*CRTC1*, *SLC27A1*, *CERS1*
	Sutai	DIAS0002715	25	SSC2: 70,246,409	14.26	1.17 × 10^-12^	*SMARCA4*, *LDLR*,
HDL-C/LDL-C	Sutai	MARC0002082	1	SSC2: 76265092	7.34	4.43 × 10^-7^	*GNA11, ABCA7*

^*a*^.The number of genome-wide significant SNPs within the QTL regions.

^*b*^.Positions of the top SNPs on the *Sus Scrofa* Build 10.2 assembly.

^*c.*^Phenotypic variance explained by the top SNPs.

^*d*^.The candidate genes were searched from annotated genes with functional relevance to serum lipids in an interval of 5.0 Mb centered at the top SNP at each significant locus. Undetected, no apparent candidate gene was detected in the corresponding genomic locus.

**Figure 1:**
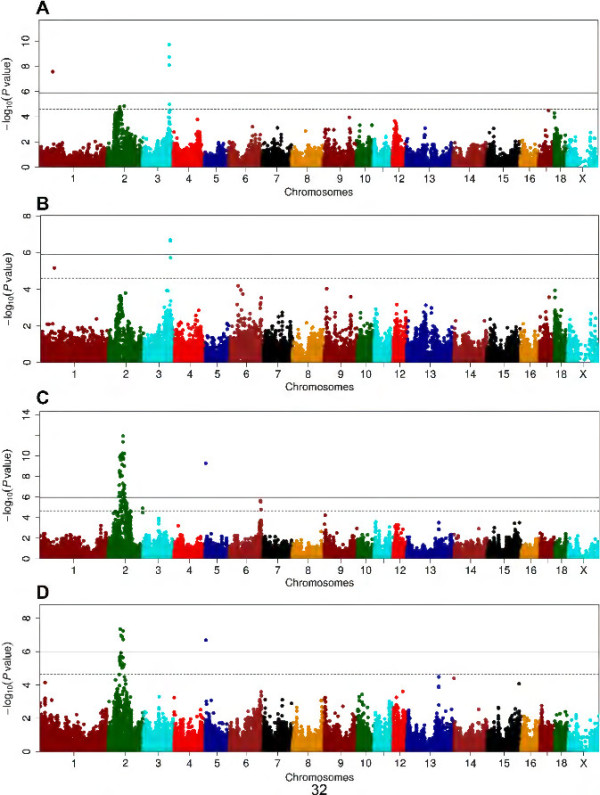
**Manhattan plots of genome-wide association analyses for serum LDL-C and TC level.** X-axis shows chromosomal positions. Y-axis shows –log10 P-values from a mixed model adjusted for sex and batch. The horizontal solid and dotted lines indicate the thresholds of genome-wide and suggestive significance levels, respectively. **(A)** and **(B)**, respectively, for LDL-C and TC in the White Duroc × Erhualian F_2_ population; and **(C)** and **(D)** for LDL-C and TC in the Sutai pigs.

We found 4 SNPs associated with TC in this resource population, of which 3 were mapped to the region around 125.00 Mb on SSC3 and achieved genome-wide significance level (Figure 1). This genomic locus is responsible for 7.60% of phenotypic variation, and *APOB* in the region is an important candidate gene (Table 2). Another TC-associated SNP (*P* = 6.91 × 10^-6^) was located at SSC1: 63,541,683 bp. For HDL-C/LDL-C, only one SNP (ASGA0016328) at SSC3: 124,769,847 bp showed significant association (*P* = 1.10 × 10^-5^). A total of 21 SNPs in 4 chromosomal regions showed signals of associations with LDL-C. The most significant association was found at SSC3: 124,769,847 bp (*P* = 1.90 × 10^-10^). Another 3 LDL-C associated SNPs located near this region (Figure 1). The most number of LDL-C-associated SNPs were identified at SSC2: 55.20-78.91 Mb (n = 14), but none of them reached genome-wide significance level. The SNP ASGA0090960 at SSC1: 63,541,683 bp was also associated with LDL-C at the genome-wide significance level (*P* = 2.69 × 10^-8^). Two significant SNPs for this trait can’t be placed to the current genome assembly of Sscrofa 10.2. For TG, only one SNP at SSC4: 119,869,765 bp (ALGA0028032) showed association in this F_2_ population (*P* = 1.61 × 10^-5^, Additional file 2: Figure S2). With regard to serum HDL-C level, we observed an obvious peak of –log (*P*-value) for the SNPs near the gene cluster of *APOA5-APOA4-APOA3-APOA1* (< 1.00 Mb) (Additional file 2: Figure S2) although its associations did not achieved significance level (*P* = 3.50 × 10^-5^).

### GWAS for blood lipids in Sutai pigs

The Q-Q plots for the distribution of *P* values involving the 45,322 SNPs in Sutai pigs are shown in Additional file 1: Figure S1. Just like in the F_2_ resource population, the distributions of observed *P*-values did not deviate from null distribution. Setting *P* ≤ 2.21 × 10^-5^ (1/45,322) as the suggestive significance threshold, we totally identified 91 SNPs corresponding to 8 chromosomal regions that were associated with one or more blood lipid traits (Additional file 3: Table S1B). Of these 91 SNPs, 55 achieved the genome-wide significance threshold of *P* ≤ 1.10 × 10^-6^ (0.05/45,322), Except for SNP ALGA0109254 on SSC5, all genome-wide significant associations were detected on SSC2 (Additional file 2: Figure S2).

**Figure 2:**
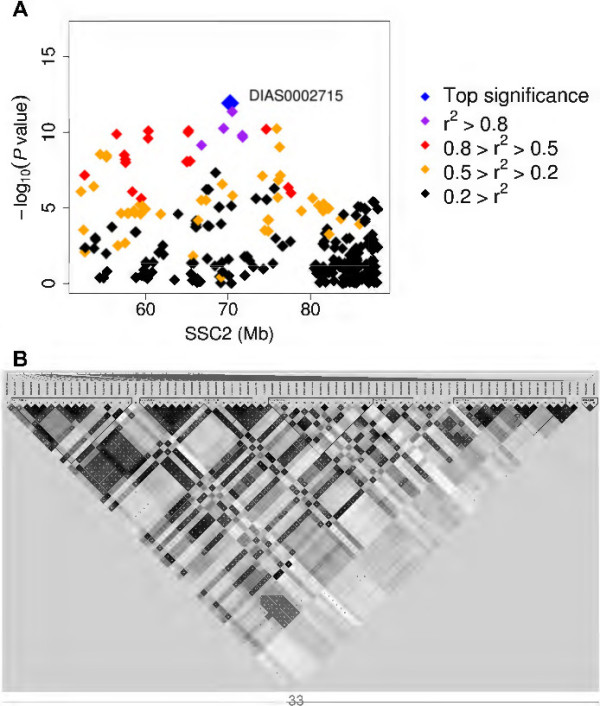
**The significant region associated with LDL-C on chromosome 2 in the Sutai pigs. (A)** Regional plot of the 36.1-Mb region from ALGA0122920 to ASGA0101845 in the Sutai pigs. Diamonds represent the significance of associations measured by –log (*P*-values), and are plotted against genomic positions on the X-axis. The colored diamonds indicate different LD between the top SNP (DIAS0002715) and other SNPs. **(B)** A LD heatmap of the 42 SNPs at genome-wide significance level in the Sutai pigs.

We found 86 SNPs in 6 chromosomal regions associated with LDL-C, including 45 genome-wide significant SNPs (Figure 1, Table 2 and Additional file 3: Table S1B). Of the 86 SNPs, 72 SNPs belonging to 4 haplotypes are located at the region from 52.14 Mb to 88.20 Mb on SSC2 (Figure 2). There were other 2 LDL-C-associated SNPs at SSC2: 160,802,237 bp and 160,802,263 bp. Three suggestive significance SNPs for LDL-C were also observed at SSC6: 145.60-147.92 Mb (*P* < 1.63 × 10^-5^). For the chromosomal region of SSC5: 3.55 Mb, only one SNP of ALGA0109254 was associated with LDL-C at genome-wide significance level (*P* = 5.21 × 10^-10^, Table 2).

A total of 28 SNPs in 3 chromosomal regions were associated with serum TC levels, including 23 SNPs at SSC2: 54.47-74.62 Mb (*P* = 9.45 × 10^-6^ to 4.40 × 10^-8^) and the SNP ALGA0109254 at SSC5: 3,550,340 bp (*P* = 2.05 × 10^-7^, Table 2). The other 4 SNPs can’t be assigned to the current pig genome assembly 10.2. Twelve SNPs in 2 chromosomal regions were associated with HDL-C/LDL-C ratios. Nine out of 12 SNPs are located at SSC2: 66.75-76.35 Mb including MARC0002082 whose association achieved the genome-wide significance level (*P* = 4.43 × 10^-7^). Significant association with HDL-C/LDL-C was also observed for SNP ALGA0005129 at SSC1: 107,283,424 bp (*P* = 1.34× 10^-5^, Additional file 2: Figure S2). Only two SNPs M1GA0008041 and MARC0094955 at SSC5: 81,575,651 bp and 82,196,866 bp were significantly associated with TG (*P* = 2.02 × 10^-5^ and 2.82 × 10^-6^).

### Quantitative trait transcripts and eQTL for blood lipids

To detect the genes whose expressions were associated with phenotypes of blood lipids, the expression levels of genome-wide transcripts in livers were determined by tag based RNA-Seq for 497 F_2_ animals. We obtained an average of 5.72 million clean tags for each animal. These clean tags were mapped to 42,121 pig transcripts. The expression data were normalized for each sample to obtain gene expression levels, and then were adjusted for gender, batch and kinship. Total 15,198 transcripts expressed in less than 97 animals (~20%) were discarded from further analysis. We used regression models to identify transcripts that were associated with each trait of blood lipids. At a significance threshold of *P* < 5 × 10^-4^, we found 58, 437, 115 and 108 transcripts whose expression levels were associated with TC, HDL-C, TG and LDL-C, respectively (Table 3 and Additional file 4: Table S2). The correlation coefficients ranged |0.18-0.42|. Total 307 transcripts were positively correlated with phenotypes and 411 were negatively correlated with phenotypes. There were 61 transcripts associated with more than one phenotype.

**Table 3:** **The number of transcripts that correlated with blood lipid traits and their characteristics of eQTL mapping**

Trait	Positive correlation	Negative correlation	Total eQTL	Cis-eQTL	Trans-eQTL
LDL-C	88	20	142	23	118
HDL-C	138	299	207	61	139
TG	41	74	169	29	139
TC	40	18	136	7	127
Total	307	411	654	120	523

To link eQTL to a phenotype of blood lipids, we focused on those transcripts that had trait-associated expression and performed GWAS using 37,540 SNPs. At a significance threshold of *P* < 1 × 10^-7^, 654 eQTL were mapped for 228 transcripts. The eQTL number for each transcript ranged from 1 to 19. No eQTL was identified for total 490 transcripts with trait-associated expressions. The identified eQTL comprised of 120 *cis*-eQTL (defined as those that mapped within 5 Mb from gene that encodes the transcript), and 523 *trans*-eQTL, those that mapped elsewhere in the genome (Table 3). The acting ways of 11 eQTL were unknown because the locations of either the SNP markers or the transcripts can not be unambiguously mapped to the current reference genome assembly (Sscrofa 10.2).

To characterize candidate genes for blood lipids, we integratively analyzed the data of GWAS, QTT and eQTL in the White Duroc × Erhualian F_2_ resource population. The genes showing concordant association signals of GWAS, QTT and eQTL are promising candidate genes. As a result, only one transcript of gnl|UG|Ssc#S35330332 encoding a hypothetical protein LOC100517809 on SSC2: 79.31 Mb for LDL-C satisfied this criterion (Additional file 3: Table S1 and Additional file 4: Table S2). Unfortunately, this transcript is poorly annotated in the current porcine genome assembly (Sscrofa 10.2).

## Discussion

To our knowledge, this study represents the first effort to identify genetic loci for serum lipids using a GWAS approach in pigs. We totally identified 109 SNPs that were significantly associated with LDL-C, TC, TG and the ratio of HDL-C/LDL-C in two experimental populations. The genomic loci of SSC2: 52.14 ~ 60.34 Mb, SSC2: 85.80 ~ 88.20 Mb, SSC6: 145.60 ~ 147.92 Mb and SSC5: 3.55 Mb were firstly identified to harbor the LDL-C associated SNPs. The most prominent locus was detected on SSC3 for LDL-C and TC in the White Duroc × Erhualian F_2_ resource population and on SSC2 for LDL-C and TC in the Sutai pigs. For several regions, such as SSC1: 63.54 Mb, SSC4: 119.87 Mb and SSC5: 3.55 Mb, only one SNP at each locus achieved the significance level (Additional file 2: Figure S2). Hence, the possibility of false positive result can not be excluded.

The SNP effects at SSC1: 107.28 Mb for HDL-C/LDL-C, SSC1: 63.54 Mb for TC and LDL-C, SSC3: 124.77-125.64 Mb for TC, LDL-C and HDL-C/LDL-C, SSC2: 66.75-74.62 Mb for TC, LDL-C and HDL-C/LDL-C, and SSC4: 119.87 Mb for TG overlapped with the previously reported QTL for blood lipids in a White Duroc × Erhualian F_2_ resource population, a commercial Duroc line and a Duroc × Pietrain F_2_ population [21, 28, 29]. However, the significant loci identified here were not well consistent with our previous QTL mapping results in the same F_2_ population [21]. For instance, the most prominent locus on SSC3 for LDL-C and TC was not identified by our previous QTL mapping. This explanation could be that: (1). there is an assumption that the QTL alleles are alternatively fixed in the founder breeds in the composite interval QTL mapping [30]. However, the GWAS were conducted without this *priori* assumption. So GWAS can detect loci at which alleles are segregating in founder animals; (2). Only additive effect was considered in the model of GWAS. However, both additive and dominant effects were included in the QTL mapping model; (3). In the QTL mapping, the detected QTL was fixed as the genetic background for next round QTL identification but no conditional analysis was performed in the present GWAS. (4). The marker density (194 microsatellite markers across the genome) is much lower in the QTL mapping compared to the GWAS.

Except that the SNPs at SSC2: 55.201-59.45 Mb and 78.91 Mb had significant associations with LDL-C in both F_2_ and Sutai populations, it was unexpected to observe distinct associations for TC, TG and HDL-C/LDL-C in Sutai and F_2_ pigs. For example, the significant association of the SNP at SSC3: 124,769,847 bp with LDL-C and TC in the White Duroc × Erhualian F_2_ resource population was not repeated in the Sutai pigs. Although the F_2_ and Sutai populations were originated from the same founder breeds of Duroc and Erhualian, the principle component analysis showed a clear divergence of the two populations (Additional file 5: Figure S3). The different association profiles in the two populations could be a result of population heterogeneity.

We found that some SNPs were associated with multiple blood lipid traits. For examples, three SNPs at SSC3: 124.77-125.64 Mb region were significantly related to LDL-C, TC and HDL-C/LDL-C in the White Duroc × Erhualian F_2_ resource population; and the SNPs harboring at SSC2: 66.75-74.62 Mb were associated with LDL-C, TC and HDL-C/LDL-C in the Sutai pigs. These QTL tend to be caused by a common variant with pleiotropic effects.

The chromosomal region of SSC2: 52.14-88.20 Mb contained the most numbers (n = 42) of genome-wide significant SNPs associated with LDL-C in the Sutai pigs (Figure 2A). To determine whether this result was caused by linkage disequilibrium (LD) between SNPs or by multiple causative genes within this region, we reconstructed haplotypes corresponding to these significant SNPs in the Sutai pigs. We found that all SNPs resided in 7 haplotype blocks (Figure 2B). Furthermore, two internal regions (60.34-64.97 Mb and 82.22-85.80 Mb) did not harbor any significant SNPs (Additional file 3: Table S1). Altogether, we assume that this chromosomal region contain multiple QTL (genes) for LDL-C. In humans, the homologous regions contain several candidate genes including *LDLR*, *SMARCA4* and *HMGCR* related to LDL-C [3, 31].

The phenotypic variance explained by the top SNPs was larger than that by the most cases of identified SNPs in human studies [3, 4]. The similar situation was also reported in dogs [32]. The possible explanations should be that: (1) the experimental population had the small effective population size. The F_2_ population used in this study was derived from two divergent pig breeds of 2 White Duroc boars and 17 Chinese Erhualian sows. (2) The environment factors could be well controlled as all experimental pigs were managed in the uniform living conditions with the same diets. (3) As for the Sutai population, the moderate sample size likely caused the inflated phenotypic variance accounted for by the SNPs [33].

We searched candidate genes with functional relevance to serum lipids or lipid metabolism in an interval of 5.0 Mb centered at the top SNP at each significant locus. The large interval was adopted as high LD extents were expected in the current experimental populations. A number of candidate genes for human blood lipids were also evidenced in this study (Table 1). *APOB*, a candidate gene for LDL-C and TC in humans [3, 4, 31] is located at SSC3: 124.77-125.64 Mp where significant associations with LDL-C and TC were observed in this study. *SMARCA4* and *LDLR* are candidates for LDL-C and TC in humans [3, 31], and their pig homologous regions were also identified SNPs that were associated with LDL-C and TC. Other candidate genes, such as *HMGCR* on chromosome 5 (HSA5) for TC and LDL-C, and *LIPG* on HSA18 for LDL-C and HDL-C [3, 4, 31], correspond to SSC2: 85.9 Mb and SSC1: 107.3 Mb for LDL-C and HDL-C/LDL-C, respectively (Additional file 3: Table S1). Besides, we identified some interesting candidate genes on the basis of knock-out mice data. For instance, SSC2: 59.75 Mb for TC encompasses the *SLC27A1* gene. *SLC27A1* knock-out mice show abnormal lipid and triglyceride levels [34]. Other potential candidate genes, such as *GNA11*, *ABCA7*, *CSF1*, *CERS1*, *SIRT3* and *SCP2*, reside in the genomic regions related to TC, LDL-C, HDL-C/LDL-C and TG in this study (Additional file 3: Table S1). These genes are associated with circulating triglyceride level (*GNA11*) [35, 36], circulating total cholesterol and HDL-C level (*ABCA7*, *CSF1*) [37, 38], abnormal lipid homeostasis (*CERS1*) [39], and circulating LDL-C level (*SIRT3*, *SCP2*) [40, 41] in knock-out mice. No apparent candidate gene was found in the genomic loci of SSC1: 63.54 Mb and SSC5: 3.55 Mb. However, as mentioned above, false positive finding can not be excluded for the regions.

To characterize more promising candidate genes, the correlations of gene expression profiles with blood lipid traits were evaluated using porcine liver samples as liver is a metabolically active organ and is critical to blood lipid metabolism. We detected functional candidate genes for these traits by exploiting phenotype-correlated expression. Some genes with trait-correlated expression in pigs appear to be the candidate genes for blood lipids in humans and mice. For examples, the expression levels of *CYP8B1*, *SCD* and *TLR2* in liver were significantly correlated with LDL-C in this study (*P* = 1.33 × 10^-5^, 3.65 × 10^-5^ and 2.11 × 10^-4^, Additional file 4: Table S2). In mice, these genes respond to the abnormal circulating cholesterol level [42–44]. *FASN*, *PPP1R3B*, *CEBPB*, *PCK1*, *APOA4*, *THRSP* and *ALMS1* are associated with the decreased circulating triglyceride level in knock-out mice [45, 46]. The expression levels of these genes were correlated with TG in this study (Additional file 4: Table S2). *IRS2*, *HIF1AN*, *CRP*, *SPTLC2*, *HSD11B1*, *SOAT1* and *SIK3* have been reported to associate with circulating HDL-C level in knock-out mice [47–49]. Their expressions were associated with HDL-C in pigs in this study (Additional file 4: Table S2).

eQTL were identified for about 31.8% of trait-correlated transcripts. Consistent with the finding in Ponsuksili et al., the proportion of *trans*-eQTL (80.0%) was higher than that of *cis*-eQTL (18.3%) [17]. In the GWAS studies in humans and mice, 10-15% of the top associated SNPs have affected a known eQTL [50]. It has been reported that trait-associated SNPs are more likely to be eQTLs [51]. However, we found only one example (gnl|UG|Ssc#S35330332) of the concordant location of eQTL, pQTL and QTT. The possible reasons for the discrepancy could be: (1) the poor annotation of current pig genome-assembly. In this study, only 61.0% of transcripts corresponding to 84.2% of clean tags can be annotated to a known gene, and about 10.0% of trait-associated transcripts can’t be placed to the current pig genome-assembly (Additional file 4: Table S2); (2) A considerable proportion of structural mutations rather than regulatory mutations in responsible genes contribute to blood lipids. For instance, protein-altering variants in *APOB* and *LDLR* cause phenotypic variation in human blood lipids [3]; and (3) Compared to human studies, our sample sizes are small and thereby lead to the failure detection of QTL with small effects, which may harbor the eQTL for trait-related transcripts.

## Conclusions

To our knowledge, this is the first study on identifying the genomic regions associated with blood lipids by an integrative analysis of GWAS, QTT and eQTL mapping in pigs. We firstly reported 4 novel genomic loci for porcine LDL-C level. Several chromosomal regions such as SSC2: 64.97-82.22 Mb and SSC3: 124.77-126.93 Mb are worthwhile for further fine-mapping and identifying the causative mutations for LDL-C and TC. Moreover, many genomic regions previously identified in humans and mice have been replicated here. A list of human or mice candidate genes were also evidenced in this study. This study would benefit the identification of causative genes for blood lipid traits and also gives useful information for studies of human cardiovascular diseases.

## Electronic supplementary material

Additional file 1: Figure S1: Quantile-quantile (Q-Q) plots of the observed *P*-values versus the expected *P*-values of association in GWAS for blood lipid. (DOC 588 KB)

Additional file 2: Figure S2: Manhattan plots of genome-wide association analyses for serum TG, HDL-C and HDL-C/LDL-C. (DOC 1 MB)

Additional file 3: Table S1: SNPs significantly associated with blood lipids in White Duroc X Erhualian F2 population and Sutai pigs. (XLS 38 KB)

Additional file 4: Table S2: Transcripts significantly associated with blood lipids and its eQTL. (XLS 163 KB)

Additional file 5: Figure S3: The PCA analysis showed a clear divergence of the two populations. (DOC 100 KB)

## Abbreviations

TC: Total cholesterol
LDL-C: Low-density lipoprotein cholesterol
HDL-C: High-density lipoprotein cholesterol
TG: Triglycerides
APOB: *apolipoprotein B*
LDLR: *LDL receptor*
QTT: Quantitative trait transcript
eQTL: Expression quantitative trait locus
SMARCA4: SWI/SNF related, matrix associated, actin dependent regulator of chromatin, subfamily a, member 4
HMGCR: 3-hydroxy-3-methylglutaryl-CoA reductase.
