# Supplementary material for: Genetic dissection of blood lipid traits by integrating genome-wide association study and gene expression profiling in a porcine model
Source: BMC Genomics. 2013 Dec 3;14(1):848. doi: 10.1186/1471-2164-14-848 (PMC4046658; doi:10.1186/1471-2164-14-848)
Supplement: Supplementary file 1 — Additional file 1: Figure S1: Quantile-quantile (Q-Q) plots of the observed P-values versus the expected P-values of association in GWAS for blood lipid. (DOC 588 KB) [file 12864_2012_5543_MOESM1_ESM.doc]

**Figure legends**

**Figure S1.** **Quantile-quantile (Q-Q) plots of the observed *P*-values versus the expected *P*-values of association in GWAS for blood lipid.**

(A) For LDL-C in F2 population; (B) For LDL-C in Sutai population; (C) For TC in F2 population; (D) For TC in Sutai population; (E) For HDL-C in F2 population; (F) For HDL-C in Sutai population; (G) For TG in F2 population; (H) For TG in Sutai population; (I) For HDL-C/LDL-C in F2 population; (J) For HDL-C/LDL-C in Sutai population.


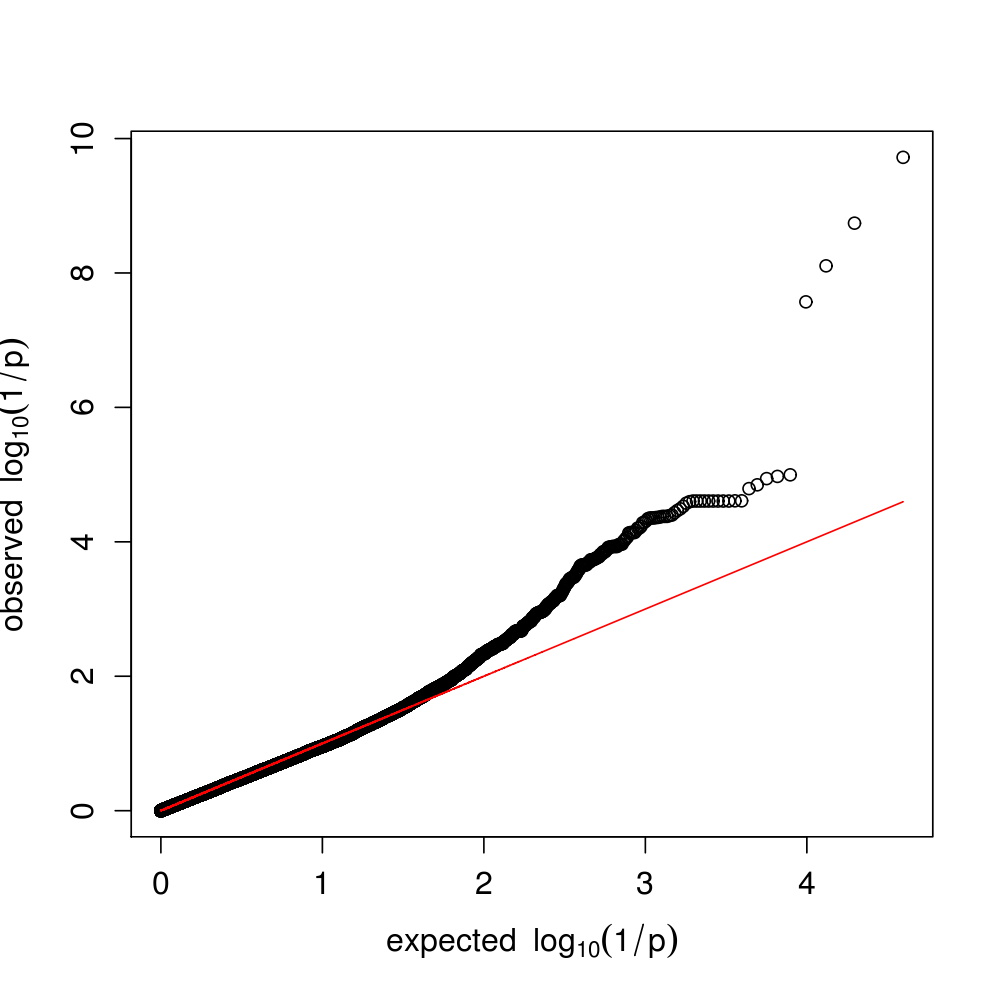

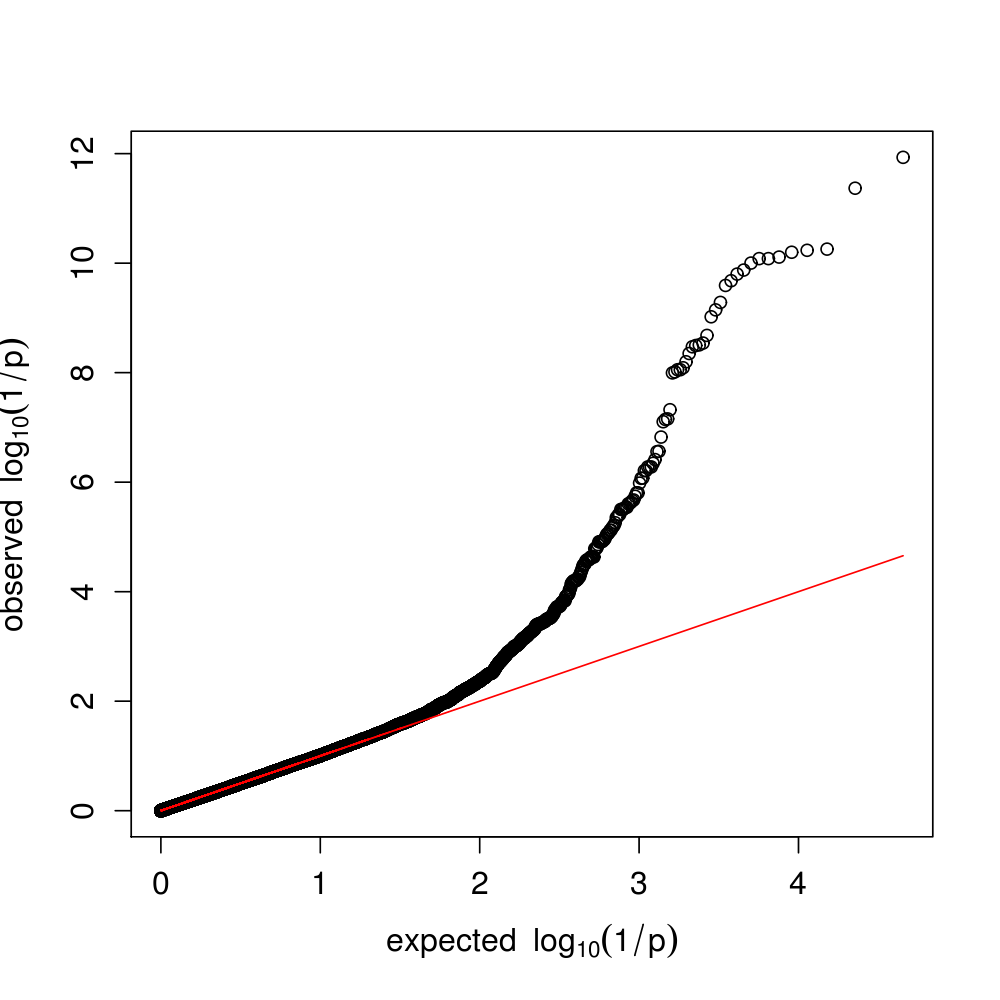


A

B


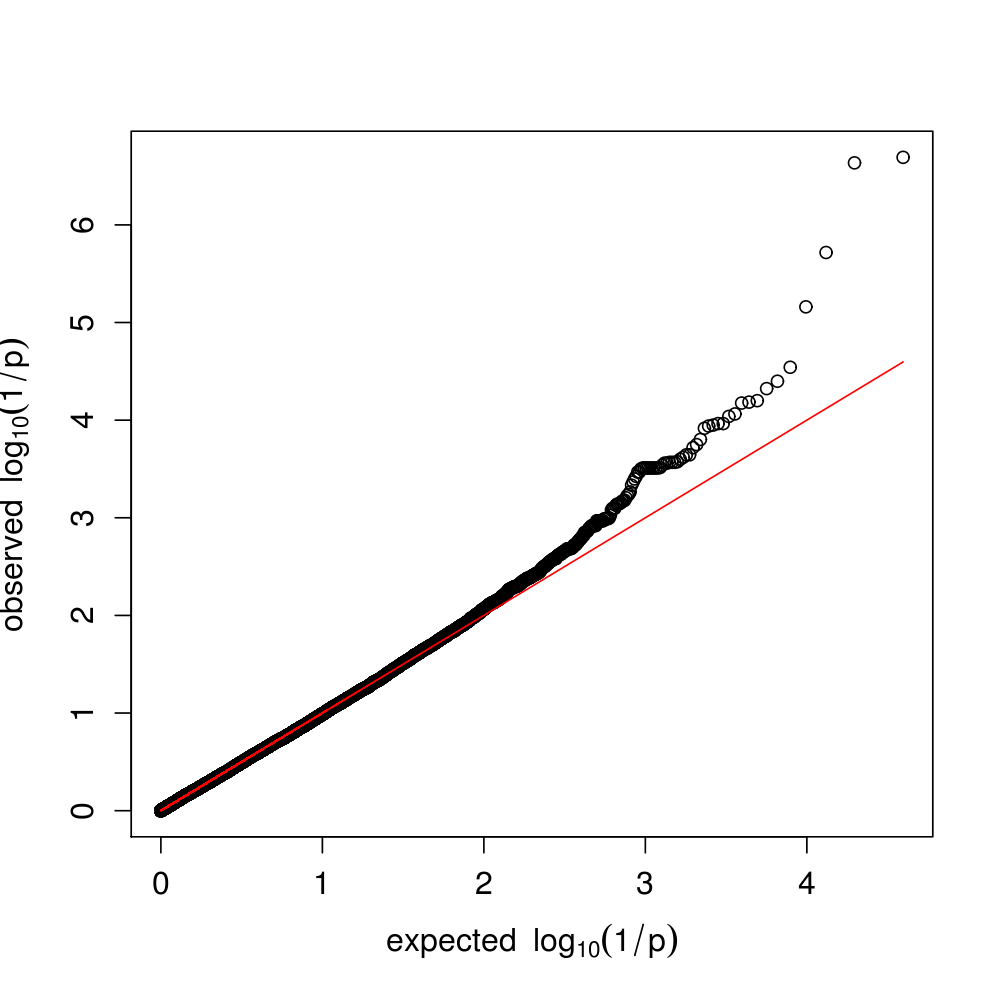

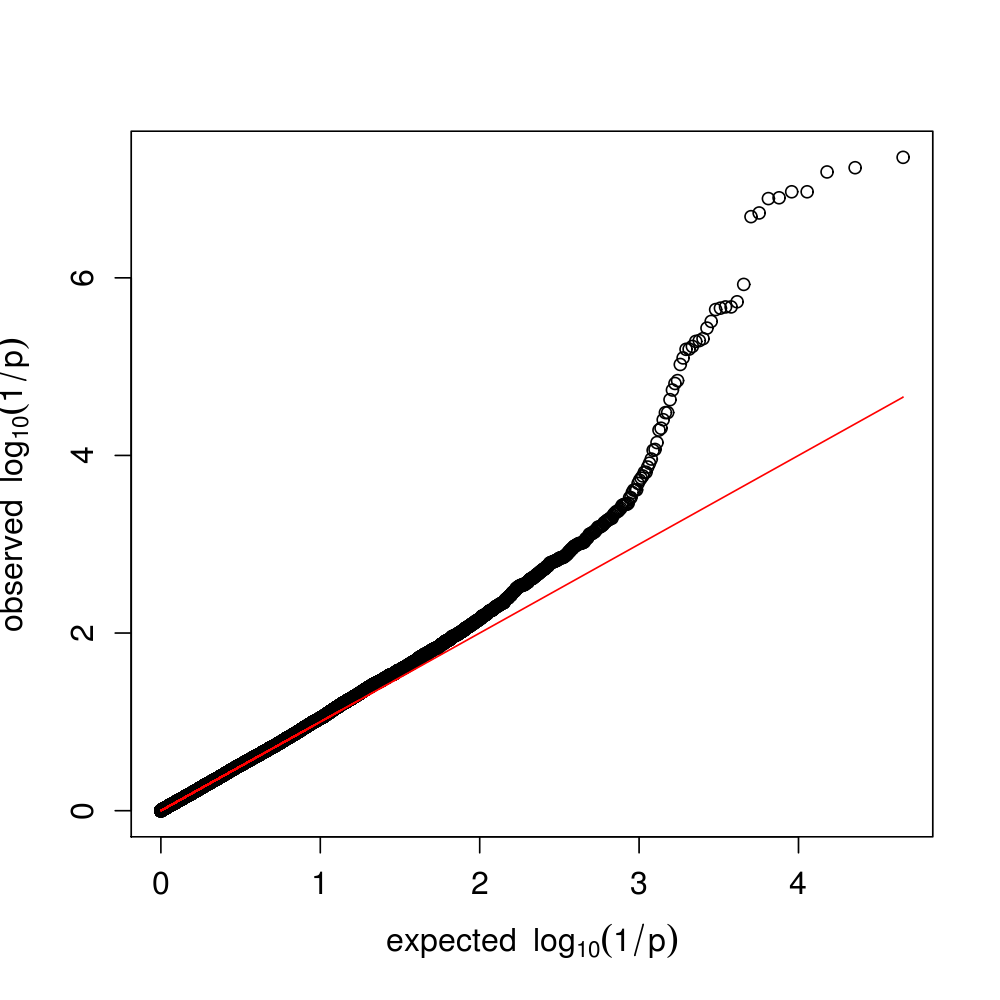


C

D


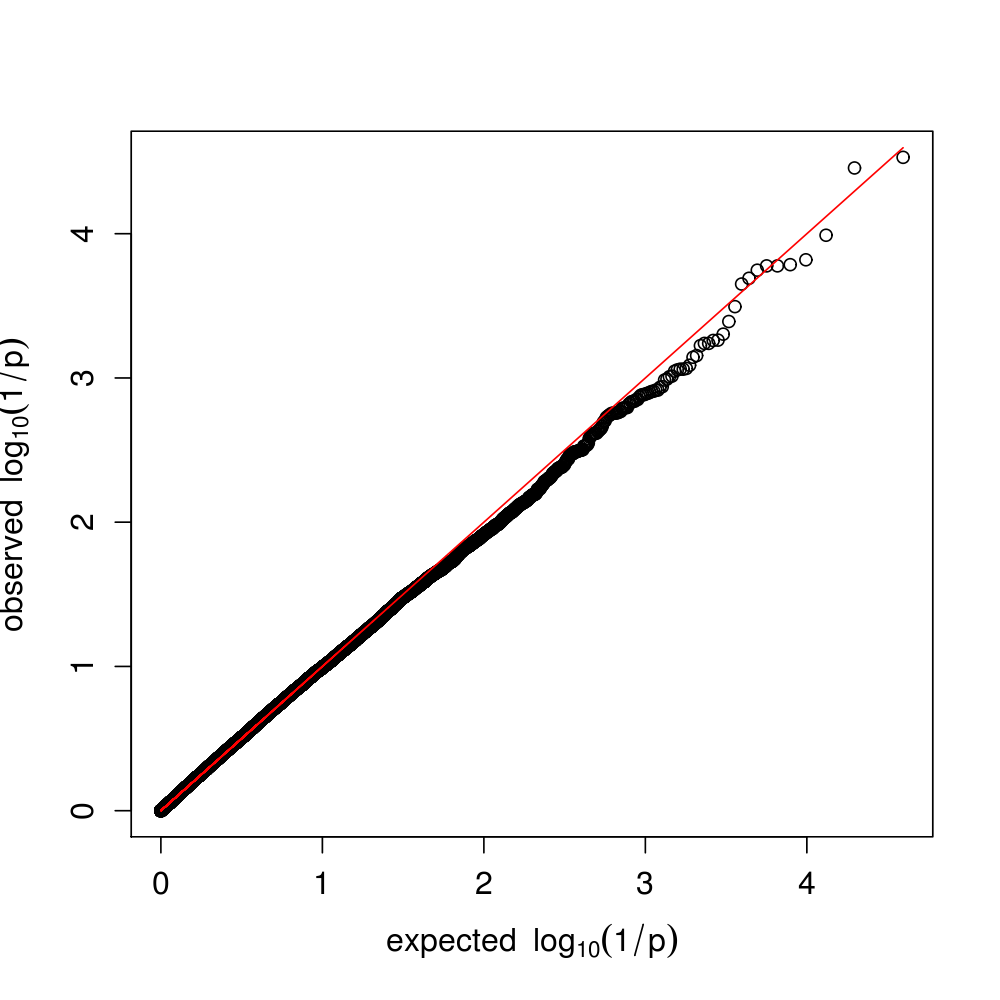

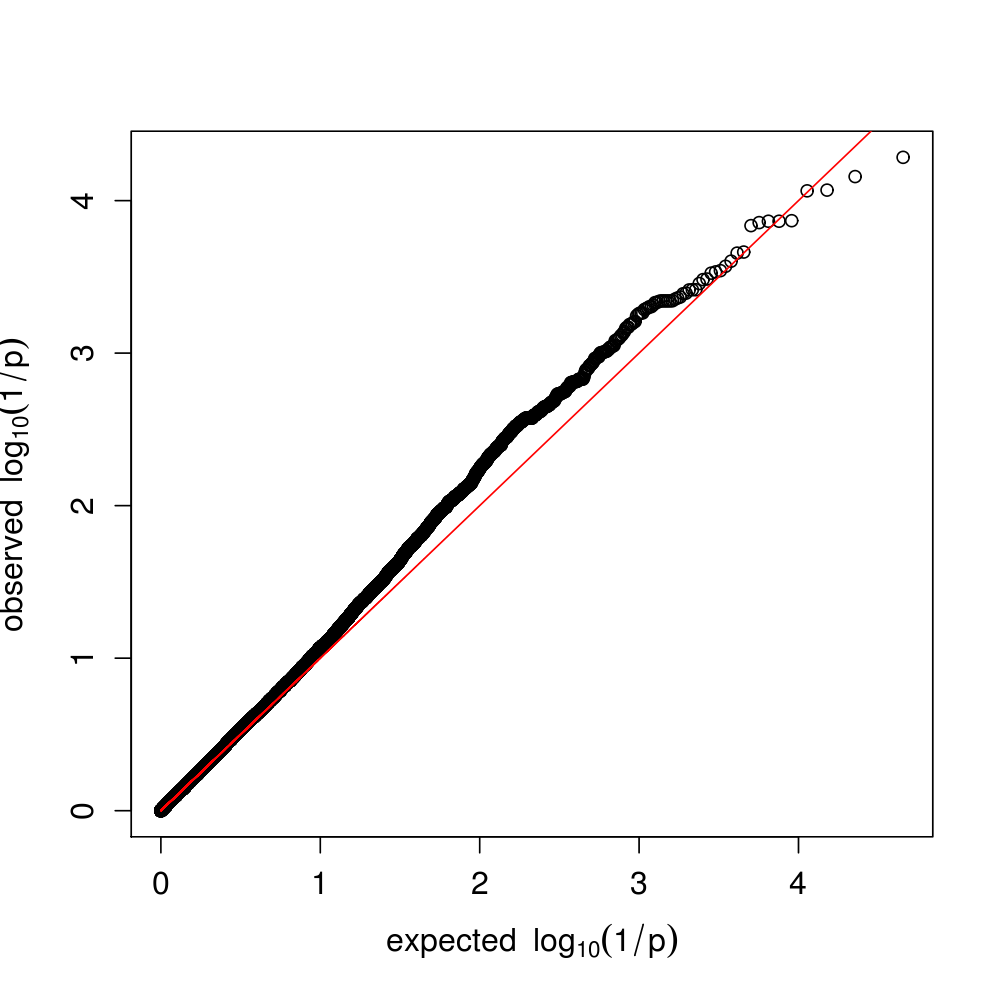


E

F


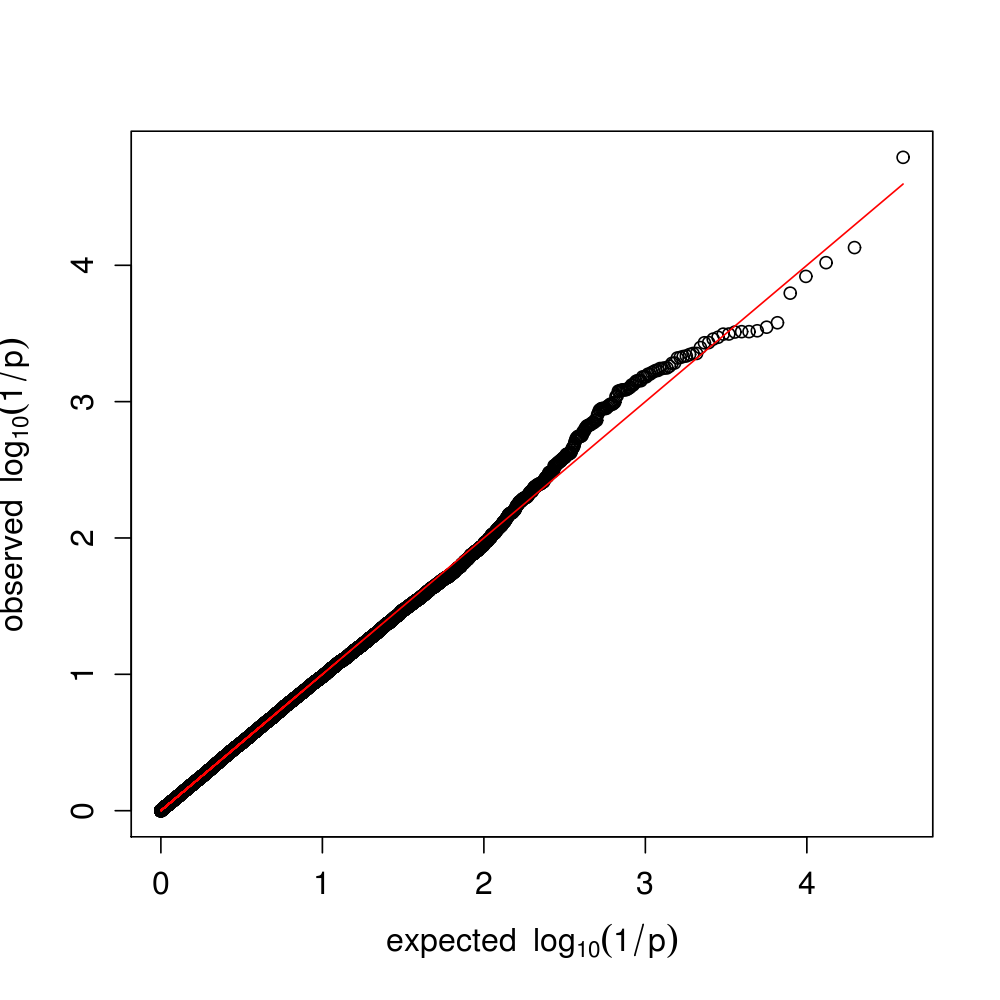

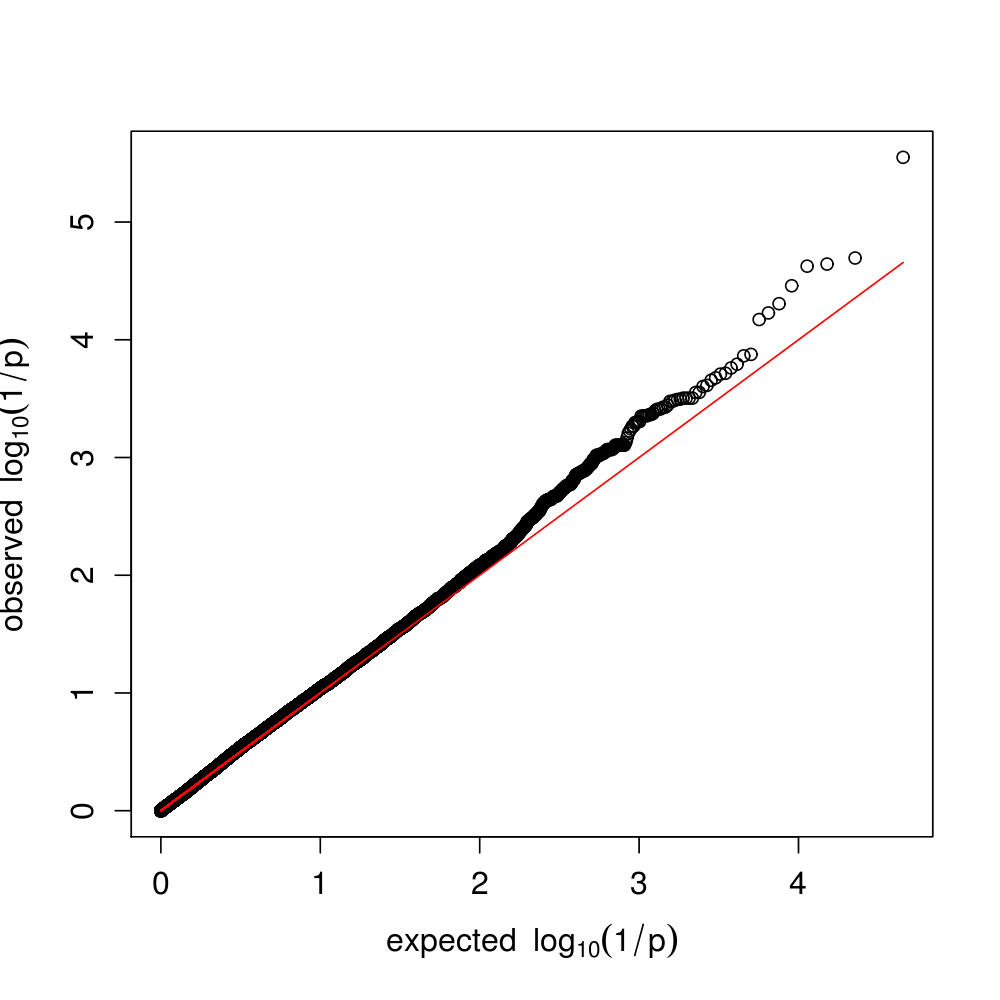


G

H


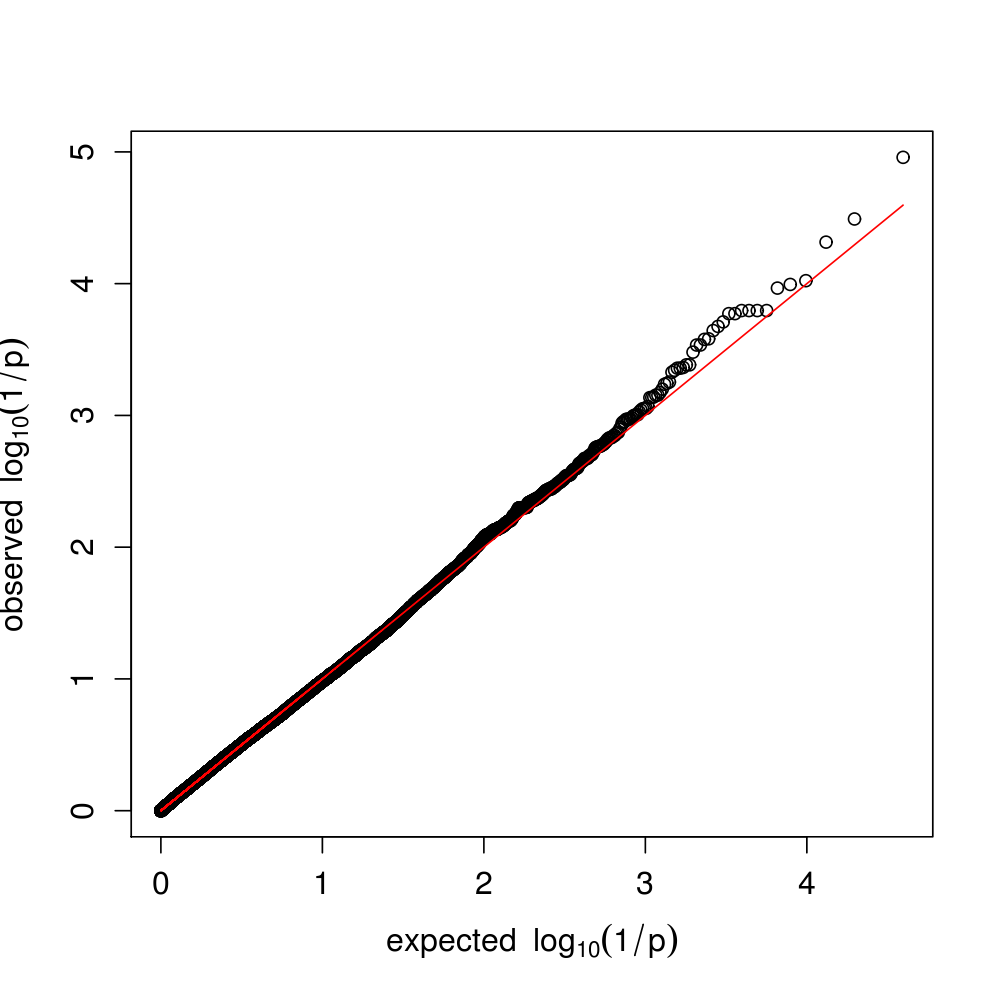

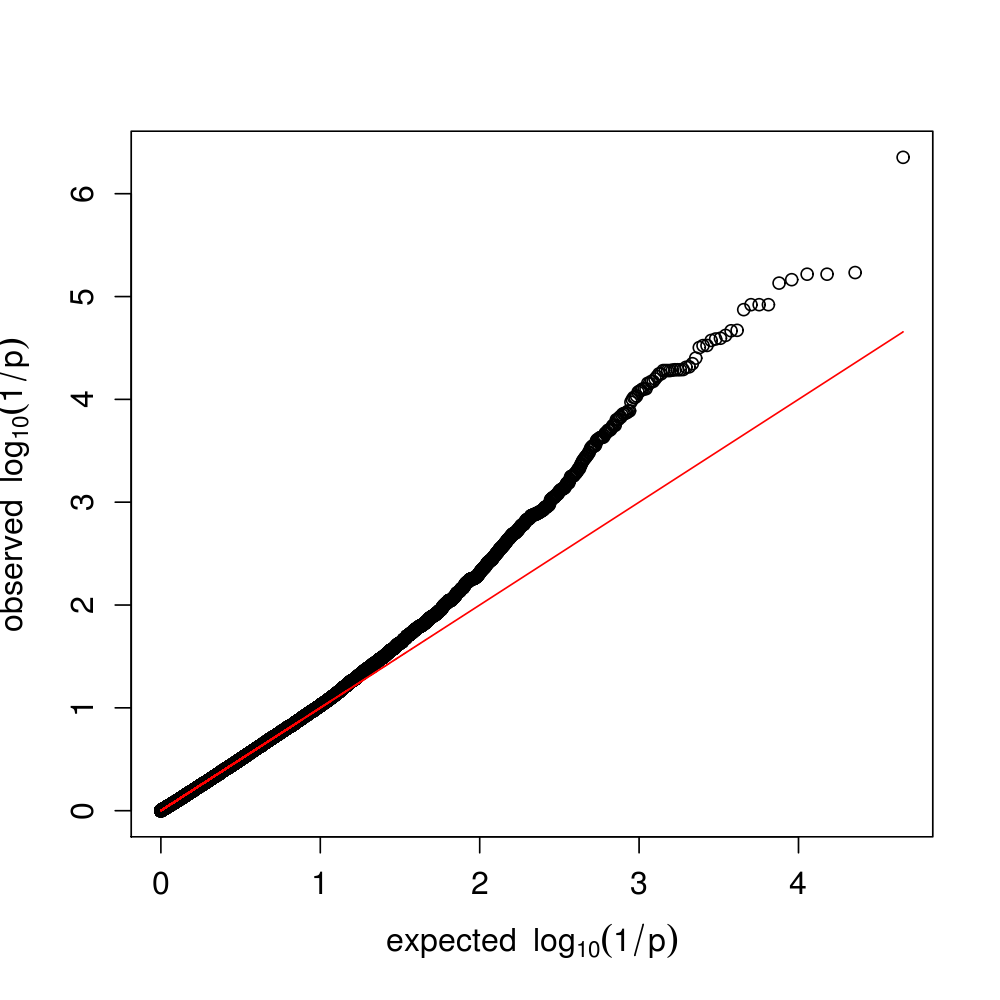


I

J
