# Supplementary material for: Genetic dissection of blood lipid traits by integrating genome-wide association study and gene expression profiling in a porcine model
Source: BMC Genomics. 2013 Dec 3;14(1):848. doi: 10.1186/1471-2164-14-848 (PMC4046658; doi:10.1186/1471-2164-14-848)

**Figure legends**

**Figure S2. Manhattan plots of genome-wide association analyses for serum TG, HDL-C and HDL-C/LDL-C.**

X-axis shows chromosomal positions. Y-axis shows –log10 P-values from a mixed model adjusted for sex and batch. The horizontal solid line indicates the preset threshold of genome-wide significance level; the horizontal dotted line indicates the preset threshold of suggestive significance level. (A) for TG in F_2_ population; (B) for TG in Sutai; (C) for HDL-C in F_2_ population; (D) for HDL-C in Sutai; (E) for HDL-C/LDL-C in F_2_ population; (F) for HDL-C/LDL-C in Sutai.

A


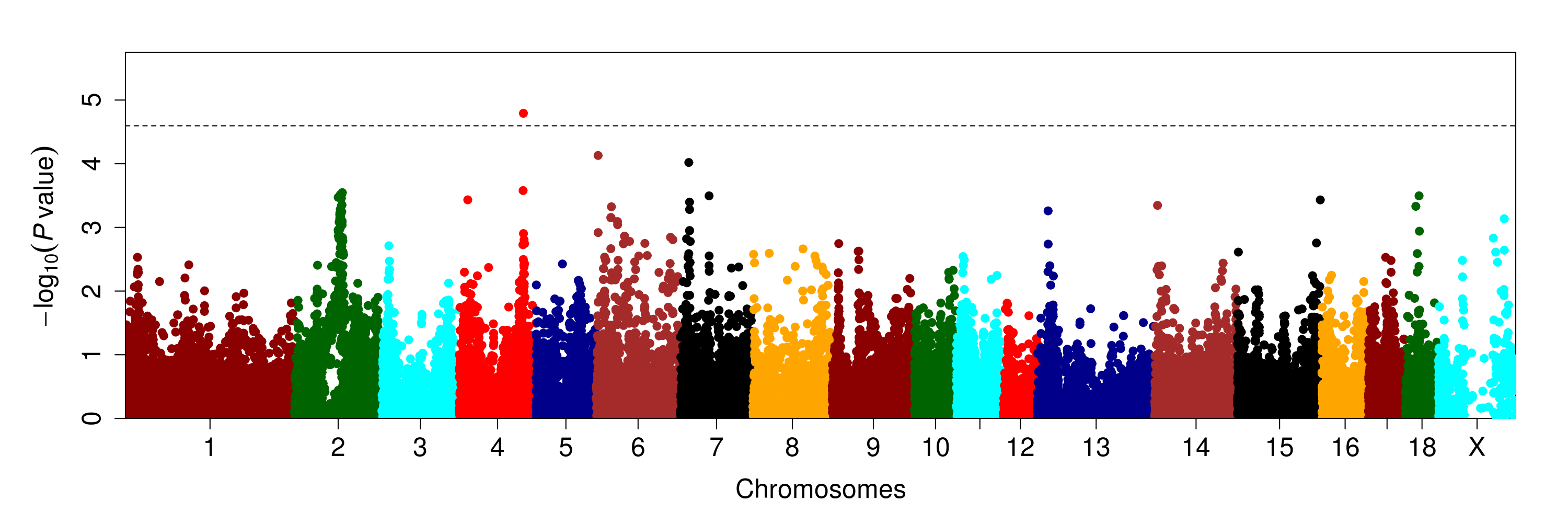


B

C


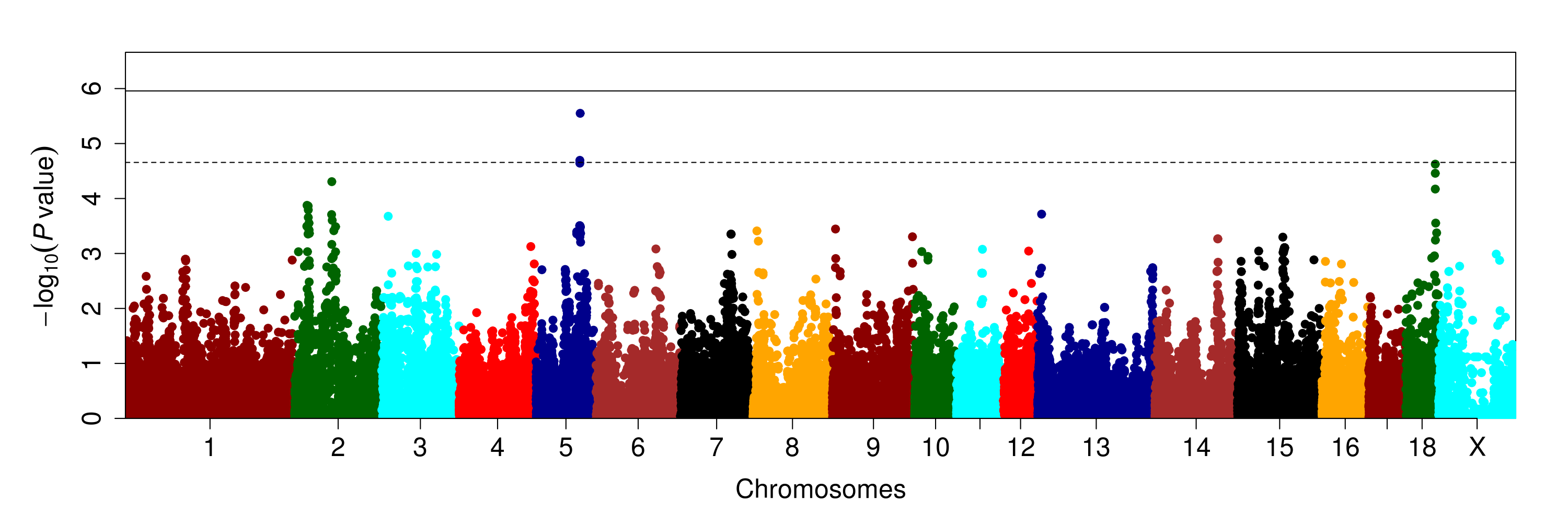


APOA5-APOA4-APOA3-APOA1 cluster


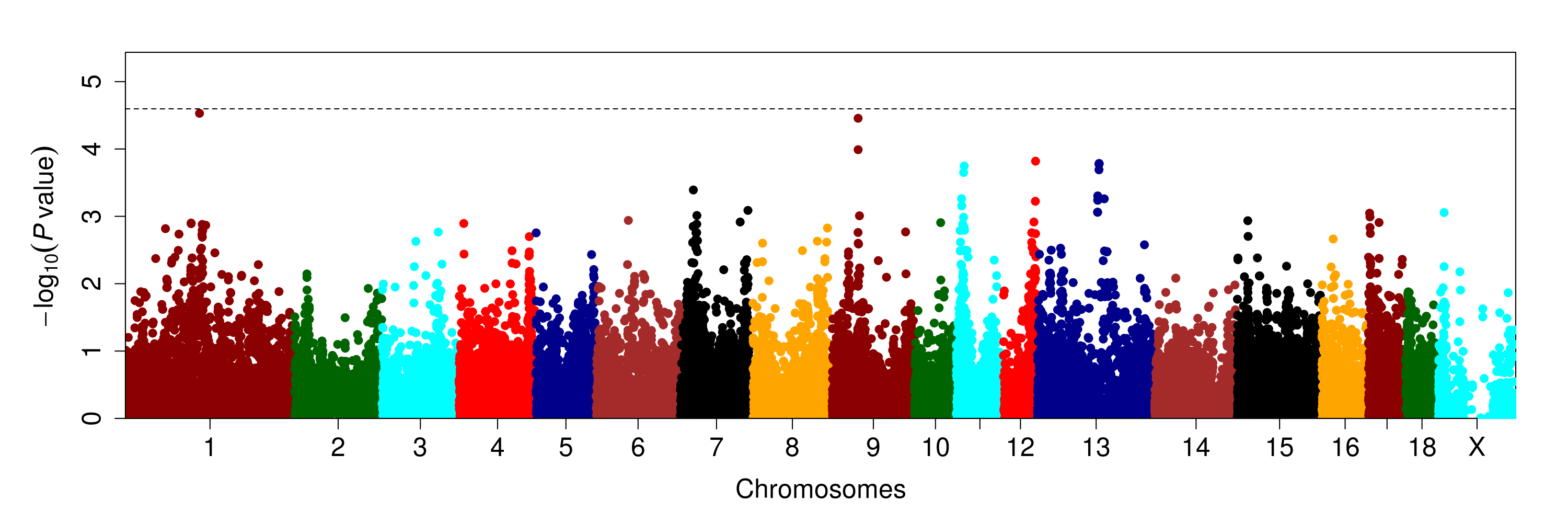


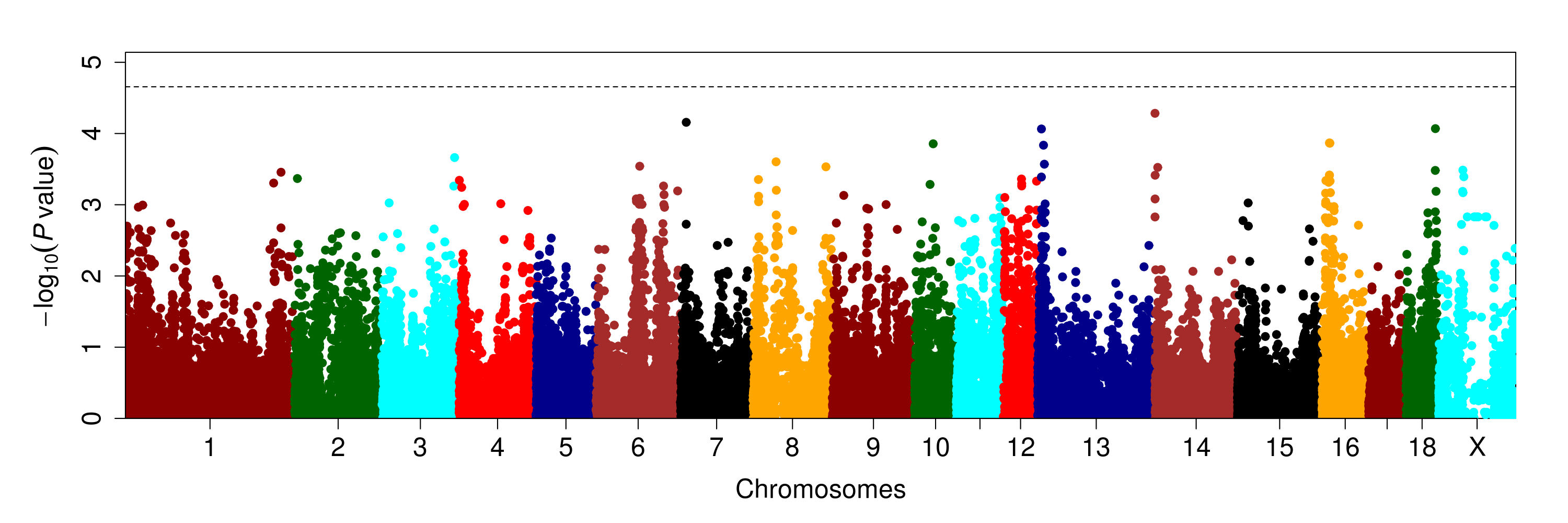


D

E


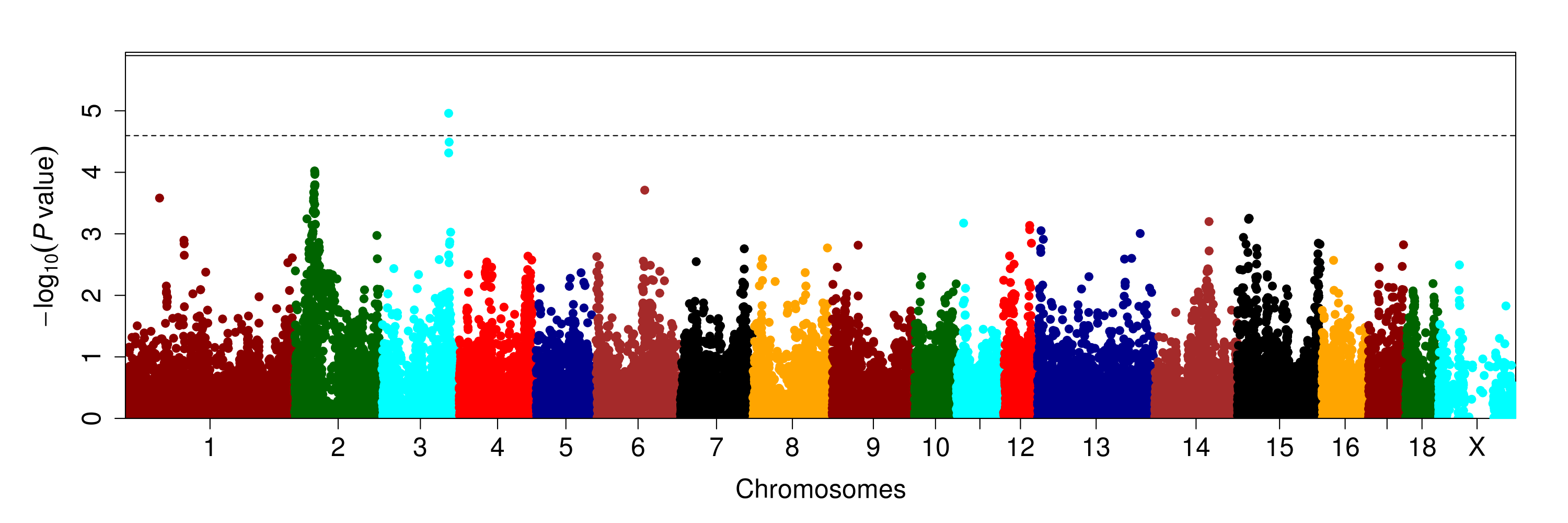


F


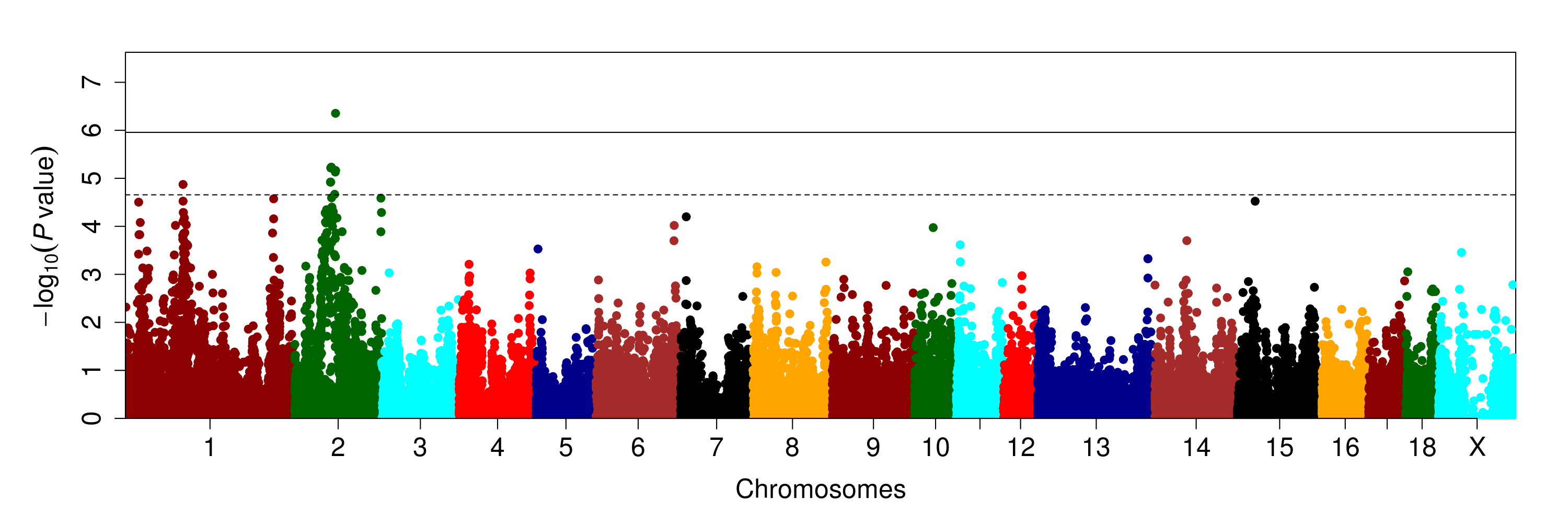

Supplement: Supplementary file 2 — Additional file 2: Figure S2: Manhattan plots of genome-wide association analyses for serum TG, HDL-C and HDL-C/LDL-C. (DOC 1 MB) [file 12864_2012_5543_MOESM2_ESM.doc]
