# Supplementary material for: Genetic dissection of blood lipid traits by integrating genome-wide association study and gene expression profiling in a porcine model
Source: BMC Genomics. 2013 Dec 3;14(1):848. doi: 10.1186/1471-2164-14-848 (PMC4046658; doi:10.1186/1471-2164-14-848)
Supplement: Supplementary file 5 — Additional file 5: Figure S3: The PCA analysis showed a clear divergence of the two populations. (DOC 100 KB) [file 12864_2012_5543_MOESM5_ESM.doc]

**Figure S3. The PCA analysis showed a clear divergence of the two populations**


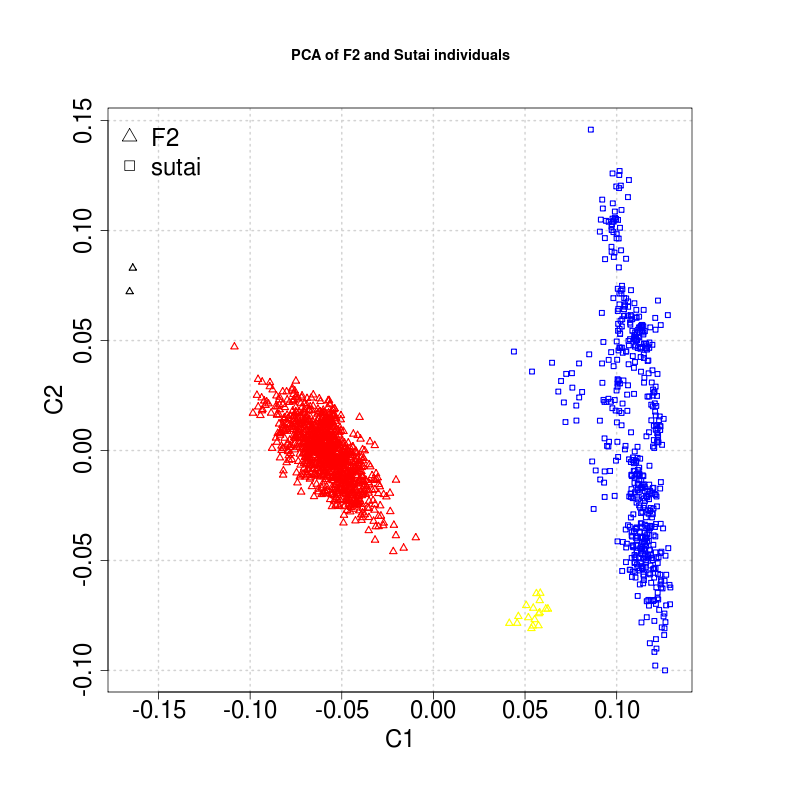


The triangles and rectangles represent the individuals from the F_2_ and Sutai population, respectively. The black, yellow, red and blue colors denote Duroc founders, Erhualian founders, F_2_ individuals and Sutai pigs.
